# Global burden of alcoholic cardiomyopathy in middle-aged men (1990–2021) and projections to 2050: a systematic analysis of the Global Burden of Disease 2021 data

**DOI:** 10.3389/fpubh.2025.1608351

**Published:** 2025-06-26

**Authors:** Yaqing Zhou, Tao Liu, Yao Meng, Zhenjun Ji, Zhongpu Chen, Genshan Ma

**Affiliations:** Department of Cardiology, Zhongda Hospital, School of Medicine, Southeast University, Nanjing, Jiangsu, China

**Keywords:** alcoholic cardiomyopathy, Global Burden of Disease, mortality, disability-adjusted life years, forecasting, epidemiology

## Abstract

**Objective:**

Alcoholic cardiomyopathy (ACM) is a severe cardiovascular condition caused by chronic excessive alcohol consumption, leading to heart failure, increased morbidity, and mortality. This study aimed to comprehensively analyze the global burden of ACM among middle-aged males from 1990 to 2021 using the Global Burden of Disease (GBD) data. Additionally, we projected future trends until 2050 using time-series forecasting models.

**Methods:**

We extracted ACM-related mortality, disability-adjusted life years (DALYs), years lived with disability (YLDs), and years of life lost (YLLs) from the GBD 2021 dataset across 204 countries and territories. Temporal trends were assessed using estimated annual percentage change (EAPC). Regional clustering was performed using hierarchical analysis. Future projections were estimated using Autoregressive Integrated Moving Average (ARIMA) and Exponential Smoothing (ES) models.

**Results:**

In 2021, ACM caused 51,494 deaths (95% UI: 44,031–56,451), with an age-standardized death rate of 1.24 per 100,000 (95% UI: 1.06–1.36). DALYs reached 1,771,812 (95% UI: 1,521,953–1,943,500), with an age-standardized rate of 41.81 per 100,000 (95% UI: 35.97–45.8). The burden was highest in Eastern Europe, Central Asia, and Latin America. Future projections suggest a potential stabilization or gradual decline in ACM burden, though mortality and DALYs remain high in some regions.

**Conclusion:**

ACM remains a major global health challenge, particularly in middle-aged males. Regional disparities highlight the need for targeted alcohol control policies and improved cardiovascular healthcare. Strengthened public health interventions are crucial to mitigating ACM-related mortality and disability.

## Introduction

Alcoholic cardiomyopathy (ACM) is a severe form of dilated cardiomyopathy caused by chronic excessive alcohol consumption, leading to progressive cardiac dilation, systolic dysfunction, and heart failure (1). Alcohol consumption is a major global health risk, contributing to approximately 3 million deaths annually and accounting for 5.3% of global mortality (2). The burden of ACM is expected to rise due to increasing alcohol consumption, particularly in low- and middle-income countries (3). Despite the well-documented association between chronic alcohol use and cardiac dysfunction, global epidemiological trends and mortality patterns remain inadequately characterized.

Previous studies have explored pathophysiological mechanisms of ACM, including alcohol-induced cardiotoxicity, mitochondrial dysfunction, oxidative stress, and myocardial fibrosis (4). Clinical research has examined its progression and associations with comorbidities such as hypertension and arrhythmias (5). However, most studies are small-scale or region-specific, lacking a comprehensive global analysis. Furthermore, variations in ACM burden across geographic regions, age groups, and sex distributions remain poorly understood, necessitating large-scale epidemiological research.

The Global Burden of Disease (GBD) study provides a comprehensive dataset covering 204 countries since 1990, systematically estimating disease burden through mortality, disability-adjusted life years (DALYs), years lived with disability (YLDs), and years of life lost (YLLs) (6). Unlike traditional studies, GBD enables comparative assessments across populations and temporal trends, offering a robust framework for ACM analysis.

This study utilized the GBD 1990–2021 dataset to conduct an extensive epidemiological analysis of ACM, focusing on global trends, regional disparities, and demographic variations. Our objectives were to quantify ACM burden in terms of mortality and DALYs, identify key demographic patterns, and assess temporal trends over the past three decades. These findings provide critical insights for public health policies, alcohol-related disease prevention strategies, and future cardiovascular health research.

## Materials and methods

### Overview

This study comprehensively analyzed the global burden of ACM among middle-aged men using the GBD 2021 database. We aimed to quantify the burden of ACM in terms of mortality, DALYs, YLDs, and YLLs at both global and regional levels. This study adhered to the Guidelines for Accurate and Transparent Health Estimates Reporting (GATHER) (7).

Data were extracted from the GBD 2021 dataset, covering 204 countries and territories and stratified by age, socio-demographic index (SDI), regional groups, which were sourced from multiple sources.

such as household surveys, vital Statistics, and other sources (8). The estimated annual percentage change (EAPC) was calculated to assess trends in disease burden. Additionally, we used Exponential Smoothing (ES) and Autoregressive Integrated Moving Average (ARIMA) models to project ACM burden until 2050.

### Study data

#### GBD database and case definition

The GBD 2021 study provides comprehensive epidemiological estimates for ACM using a systematic framework based on demographic, clinical, and cause-specific mortality data. The ICD-10 code I42.6, used to define ACM, was mapped to the GBD cause list based on standardized diagnostic algorithms developed by the Institute for Health Metrics and Evaluation (IHME). These mappings are validated through extensive cross-referencing of hospital discharge data, vital registration systems, and verbal autopsy studies. Nevertheless, in countries with limited diagnostic capabilities or poor coding accuracy, ACM may be underreported or misclassified under broader cardiomyopathy categories. GBD models attempt to adjust for such biases through data redistribution algorithms, but residual uncertainty remains, particularly in low-resource settings.

The disease burden was measured using age-standardized death rate (ASDR), age-standardized DALYs rate, age-standardized YLDs rate and age-standardized YLLs rate (per 100,000 population).

#### Population stratification

In order to assess ACM in middle-aged men burden, the patients were stratified by: Age groups (25–29, 30–34, 35–39, 40–44, 45–49, 50–54, 55–59); Geographical regions (45 GBD regions, 204 countries); Socio-Demographic Index (SDI) groups (high, high-middle, middle, low-middle, low).

#### Temporal trends and regional clustering

We analyzed trends from 1990 to 2021, assessing changes in mortality, DALYs, YLDs, and YLLs. A hierarchical clustering analysis was conducted to identify regional patterns in ACM burden.

#### Future projections (2022–2050)

To estimate future ACM burden, we employed autoregressive integrated moving average (ARIMA) model, which is suitable for time-series forecasting using past trends (9). In addition, exponential smoothing (ES) model, that captures long-term trends and seasonal variations were performed (10). ARIMA (p,d,q) models were fitted to historical data, with optimal parameters selected via Akaike Information Criterion (AIC). Exponential Smoothing models (Holt-Winters method) were used to capture long-term trends in ACM burden.

We performed out-of-sample validation to assess the predictive performance of the ARIMA and ES models. Specifically, we trained both models on data from 1990 to 2015 and tested their forecasts against observed values from 2016 to 2021. Model accuracy was evaluated using Mean Absolute Percentage Error (MAPE) and Root Mean Squared Error (RMSE). The ARIMA model achieved slightly lower MAPE and RMSE values compared to the ES model for death and YLL predictions, indicating higher accuracy in capturing abrupt trend shifts. The ES model performed better for smoothed DALY projections, suggesting complementary strengths of the two approaches and justifying their combined inclusion in this study.

### Statistical analysis

#### Trend analysis and EAPC calculation

The EAPC was used to quantify trends in ACM burden, calculated as: EAPC = (e*β* − 1) × 100. Where *β* is the regression coefficient from a log-linear model fitted to the annual ASR (11).

#### Hierarchical clustering for regional comparisons

We applied hierarchical clustering using Ward’s method and Euclidean distance to classify GBD regions based on their ACM burden patterns (12).

#### Calculation for uncertainty intervals

Uncertainty intervals (UIs) for GBD burden estimates were derived using 1,000 draws from posterior distributions generated through Bayesian meta-regression modeling (DisMod-MR 2.1). For forecasting, UIs were generated by simulating 1,000 replicates from the residual variance structure of the fitted ARIMA and ES models, accounting for parameter uncertainty and data variability.

### Statistical software

All analyses were performed using R software (version 4.2.1) and Python (version 3.9). Statistical significance was set at *p* < 0.05 where applicable.

## Results

### The disease burden attributable to ACM in 2021

In 2021, the number of ACM-related deaths was 51,494 [95% uncertainty intervals (UI: 44,031–56,451)]. The corresponding age-standardized deaths rate was 1.24 (95% UI: 1.06–1.36) per 100,000 population (Table 1). The number of ACM-related DALYs was 1,771,812 (95% UI: 1,521,953–1,943,500). The corresponding ASR of DALYs was 41.81 (95% UI: 35.97–45.8) per 100,000 population (Table 2). The number of ACM-related YLDs was 35,784 (95% UI: 23,885–49,677). The corresponding ASR of YLDs was 0.86 (95% UI: 0.57–1.19) per 100,000 population (Table 3). The number of ACM-related YLLs was 1,736,028 (95%UI: 1,484,390–1,905,162). The corresponding ASR of YLLs was 40.95 (95% UI, 35.08–44.92) per 100,000 population (Table 4).

**Table 1:** The number of deaths cases and the age-standardized deaths rate attributable to ACM in 1990 and 2021, and its trends from 1990 to 2021 globally.

Characteristics	1990		2021		1990–2021
	Number of deaths cases (95% UI)	The age-standardized deaths rate/100000 (95% UI)	Number of deaths cases (95% UI)	The age-standardized deaths rate/100000 (95% UI)	EAPC (95% CI)
Global	33,782 (31055–36,598)	1.76 (1.63–1.91)	51,494 (44031–56,451)	1.24 (1.06–1.36)	−0.77 (−1.51–0.02)
Sex					
Male	33,782 (31055–36,598)	1.76 (1.63–1.91)	51,494 (44031–56,451)	1.24 (1.06–1.36)	−1.37 (−2.34–0.38)
Age					
25–29	630 (564–689)	0.28 (0.25–0.31)	486 (416–561)	0.16 (0.14–0.19)	−1.26 (−3.1–0.63)
30–34	1,335 (1192–1,464)	0.68 (0.61–0.75)	1,662 (1483–1871)	0.54 (0.49–0.61)	−0.66 (−2.11–0.82)
35–39	2,133 (1870–2,349)	1.19 (1.05–1.31)	3,099 (2719–3,448)	1.09 (0.96–1.22)	−0.96 (−2.06–0.16)
40–44	2,698 (2342–2,996)	1.84 (1.6–2.05)	4,397 (3897–4,878)	1.74 (1.55–1.93)	−1.42 (−2.75–0.07)
45–49	3,145 (2713–3,536)	2.66 (2.29–2.99)	5,523 (4729–6,171)	2.32 (1.99–2.59)	−1.2 (−2.85–0.49)
50–54	4,770 (4214–5,313)	4.43 (3.91–4.94)	6,287 (5146–7,151)	2.83 (2.32–3.22)	−1.26 (−2.72–0.23)
55–59	4,473 (3952–4,983)	4.82 (4.25–5.36)	7,148 (5925–7,987)	3.67 (3.04–4.1)	−0.87 (−1.93–0.19)
SDI regions					
Low SDI	222 (37–493)	0.17 (0.03–0.38)	381 (64–955)	0.14 (0.02–0.34)	−0.7 (−0.78–0.63)
Low-middle SDI	1,142 (611–1941)	0.32 (0.17–0.56)	2021 (1028–3,685)	0.26 (0.13–0.49)	−0.87 (−1–0.74)
Middle SDI	1962 (1450–2,719)	0.33 (0.24–0.46)	3,421 (1822–4,751)	0.26 (0.14–0.36)	−1.08 (−1.23–0.92)
High-middle SDI	19,120 (17282–21,138)	4.11 (3.73–4.54)	32,937 (28551–36,939)	3.74 (3.25–4.18)	−0.69 (−2.02–0.65)
High SDI	11,284 (10409–12,070)	2.42 (2.24–2.59)	12,630 (11627–13,463)	1.43 (1.32–1.52)	−1.82 (−2.08–1.57)

UI, uncertainty intervals; CI, confidence interval; SDI, socio-demographic index; EAPC, estimated annual percentage change.

**Table 2:** The number of DALYs cases and the age-standardized DALYs rate attributable to ACM in 1990 and 2021, and its trends from 1990 to 2021 globally.

Characteristics	1990		2021		1990–2021
	Number of DALYs cases (95% UI)	The age-standardized DALYs rate/100000 (95% UI)	Number of DALYs cases (95% UI)	The age-standardized DALYs rate/100000 (95% UI)	EAPC (95% CI)
Global	1,177,925 (1071258–1,275,728)	54.88 (50.18–59.4)	1,771,812 (1521953–1,943,500)	41.81 (35.97–45.8)	−0.74 (−1.56–0.08)
Sex					
Male	1,177,925 (1071258–1,275,728)	54.88 (50.18–59.4)	1,771,812 (1521953–1,943,500)	41.81 (35.97–45.8)	−1.21 (−2.33–0.07)
Age					
25–29	40,938 (36836–44,628)	18.4 (16.55–20.06)	31,842 (27549–36,576)	10.71 (9.26–12.3)	−1.24 (−3.05–0.61)
30–34	78,588 (70444–85,811)	40.24 (36.07–43.93)	97,401 (87236–109,452)	31.88 (28.55–35.82)	−0.66 (−2.1–0.8)
35–39	114,256 (100193–125,304)	63.9 (56.04–70.08)	165,682 (145599–184,046)	58.53 (51.44–65.02)	−0.95 (−2.05–0.15)
40–44	131,389 (114559–146,108)	89.84 (78.33–99.9)	213,186 (189205–236,269)	84.54 (75.03–93.7)	−1.42 (−2.73–0.08)
45–49	136,721 (118044–153,496)	115.48 (99.7–129.65)	241,564 (207805–269,275)	101.56 (87.36–113.21)	−1.19 (−2.84–0.47)
50–54	185,194 (164129–205,780)	172.03 (152.46–191.15)	244,699 (201254–277,587)	110.23 (90.66–125.05)	−1.24 (−2.7–0.23)
55–59	151,262 (133619–168,087)	162.84 (143.85–180.96)	242,443 (202104–270,985)	124.51 (103.79–139.16)	−0.86 (−1.92–0.2)
SDI regions					
Low SDI	8,098 (1754–17,323)	5.67 (1.19–12.22)	14,249 (3275–34,438)	4.43 (0.98–10.7)	−0.87 (−0.94–0.8)
Low-middle SDI	42,234 (23333–69,301)	10.75 (5.85–17.97)	73,497 (40400–127,923)	8.85 (4.76–15.62)	−0.83 (−0.97–0.69)
Middle SDI	75,503 (56309–104,045)	11.28 (8.41–15.6)	119,229 (64118–161,829)	8.59 (4.63–11.66)	−1.22 (−1.4–1.04)
High-middle SDI	719,006 (648349–792,759)	141.06 (127.29–155.62)	1,201,785 (1046653–1,346,640)	138.73 (121.21–155.03)	−0.58 (−2.01–0.88)
High SDI	331,454 (308806–350,105)	68.96 (64.24–72.87)	360,031 (334410–381,515)	43.83 (40.82–46.32)	−1.52 (−1.78–1.26)

DALYs, disability-adjusted-life-years; UI, uncertainty intervals; CI, confidence interval; SDI, socio-demographic index; EAPC, estimated annual percentage change.

**Table 3:** The number of YLDs cases and the age-standardized YLDs rate attributable to ACM in 1990 and 2021, and its trends from 1990 to 2021 globally.

Characteristics	1990		2021		1990–2021
	Number of YLDs cases (95% UI)	The age-standardized YLDs rate/100000 (95% UI)	Number of YLDs cases (95% UI)	The age-standardized YLDs rate/100000 (95% UI)	EAPC (95% CI)
Global	21,363 (14330–29,767)	0.97 (0.65–1.35)	35,784 (23885–49,677)	0.86 (0.57–1.19)	0.25 (0.03–0.48)
Sex					
Male	21,363 (14330–29,767)	0.97 (0.65–1.35)	35,784 (23885–49,677)	0.86 (0.57–1.19)	−0.17 (−0.41–0.08)
Age					
25–29	1,498 (842–2,295)	0.67 (0.38–1.03)	1,455 (830–2,313)	0.49 (0.28–0.78)	−0.51 (−0.79–0.23)
30–34	1,548 (851–2,472)	0.79 (0.44–1.27)	1714 (941–2,793)	0.56 (0.31–0.91)	−0.73 (−0.92–0.54)
35–39	1707 (987–2,648)	0.95 (0.55–1.48)	2,116 (1212–3,341)	0.75 (0.43–1.18)	−0.84 (−0.95–0.73)
40–44	1954 (1112–3,098)	1.34 (0.76–2.12)	2,696 (1502–4,319)	1.07 (0.6–1.71)	−0.91 (−1.09–0.74)
45–49	2,360 (1456–3,523)	1.99 (1.23–2.98)	4,045 (2482–6,161)	1.7 (1.04–2.59)	−0.57 (−0.92–0.22)
50–54	2,870 (1803–4,550)	2.67 (1.68–4.23)	4,372 (2654–6,862)	1.97 (1.2–3.09)	−0.4 (−0.79–0)
55–59	1708 (1000–2,732)	1.84 (1.08–2.94)	3,403 (1957–5,528)	1.75 (1–2.84)	0.33 (−0.02–0.68)
SDI regions					
Low SDI	509 (322–770)	0.3 (0.19–0.45)	1,261 (784–1880)	0.32 (0.2–0.47)	0.17 (0.15–0.19)
Low-middle SDI	951 (620–1,350)	0.22 (0.15–0.32)	1938 (1271–2,808)	0.22 (0.15–0.32)	−0.12 (−0.17–0.06)
Middle SDI	1,607 (1051–2,282)	0.22 (0.15–0.31)	3,952 (2582–5,693)	0.29 (0.19–0.42)	1.09 (0.94–1.25)
High-middle SDI	8,732 (5920–12,223)	1.7 (1.15–2.37)	12,409 (8278–17,230)	1.47 (0.98–2.04)	−0.32 (−0.56–0.08)
High SDI	9,534 (6326–13,360)	1.99 (1.33–2.78)	16,148 (10858–22,502)	2.07 (1.39–2.88)	0.41 (0.14–0.67)

YLDs, years lived with disability’s; UI, uncertainty intervals; CI, confidence interval; SDI, socio-demographic index; EAPC, estimated annual percentage change.

**Table 4:** The number of YLLs cases and the age-standardized YLLs rate attributable to ACM in 1990 and 2021, and its trends from 1990 to 2021 globally.

Characteristics	1990		2021		1990–2021
	Number of YLDs cases (95% UI)	The age-standardized YLDs rate/100000 (95% UI)	Number of YLDs cases (95% UI)	The age-standardized YLDs rate/100000 (95% UI)	EAPC (95% CI)
Global	1,156,562 (1046783–1,252,856)	53.91 (49.14–58.41)	1,736,028 (1484390–1,905,162)	40.95 (35.08–44.92)	−0.76 (−1.59–0.07)
Sex					
Male	1,156,562 (1046783–1,252,856)	53.91 (49.14–58.41)	1,736,028 (1484390–1,905,162)	40.95 (35.08–44.92)	−1.23 (−2.36–0.08)
Age					
25–29	39,440 (35312–43,165)	17.72 (15.87–19.4)	30,386 (26004–35,091)	10.22 (8.75–11.8)	−1.26 (−3.11–0.62)
30–34	77,040 (68802–84,504)	39.44 (35.23–43.27)	95,687 (85360–107,762)	31.32 (27.94–35.27)	−0.66 (−2.11–0.82)
35–39	112,549 (98648–123,903)	62.95 (55.18–69.3)	163,565 (143497–181,947)	57.78 (50.69–64.28)	−0.95 (−2.06–0.16)
40–44	129,435 (112330–143,719)	88.5 (76.8–98.27)	210,490 (186580–233,528)	83.47 (73.99–92.61)	−1.42 (−2.75–0.07)
45–49	134,361 (115876–151,034)	113.49 (97.87–127.57)	237,519 (203368–265,346)	99.86 (85.5–111.55)	−1.2 (−2.86–0.48)
50–54	182,324 (161065–203,051)	169.36 (149.61–188.61)	240,327 (196731–273,376)	108.26 (88.63–123.15)	−1.26 (−2.73–0.23)
55–59	149,553 (132119–166,605)	161 (142.24–179.36)	239,040 (198100–267,089)	122.76 (101.73–137.16)	−0.88 (−1.94–0.19)
SDI regions					
Low SDI	7,589 (1299–16,798)	5.37 (0.91–11.89)	12,988 (2207–33,244)	4.12 (0.69–10.35)	−0.94 (−1.01–0.86)
Low-middle SDI	41,283 (22478–68,475)	10.52 (5.65–17.74)	71,558 (38193–125,572)	8.62 (4.52–15.4)	−0.84 (−0.98–0.71)
Middle SDI	73,896 (54594–101,909)	11.06 (8.18–15.32)	115,278 (60046–158,685)	8.3 (4.33–11.43)	−1.28 (−1.47–1.09)
High-middle SDI	710,274 (639121–783,956)	139.35 (125.51–153.99)	1,189,376 (1032936–1,334,116)	137.26 (119.57–153.56)	−0.58 (−2.03–0.89)
High SDI	321,920 (300194–340,526)	66.97 (62.48–70.87)	343,883 (318830–364,080)	41.76 (38.83–44.12)	−1.59 (−1.86–1.33)

YLLs, years of life lost; UI, uncertainty intervals; CI, confidence interval; SDI, socio-demographic index; EAPC, estimated annual percentage change.

**Figure 1:**
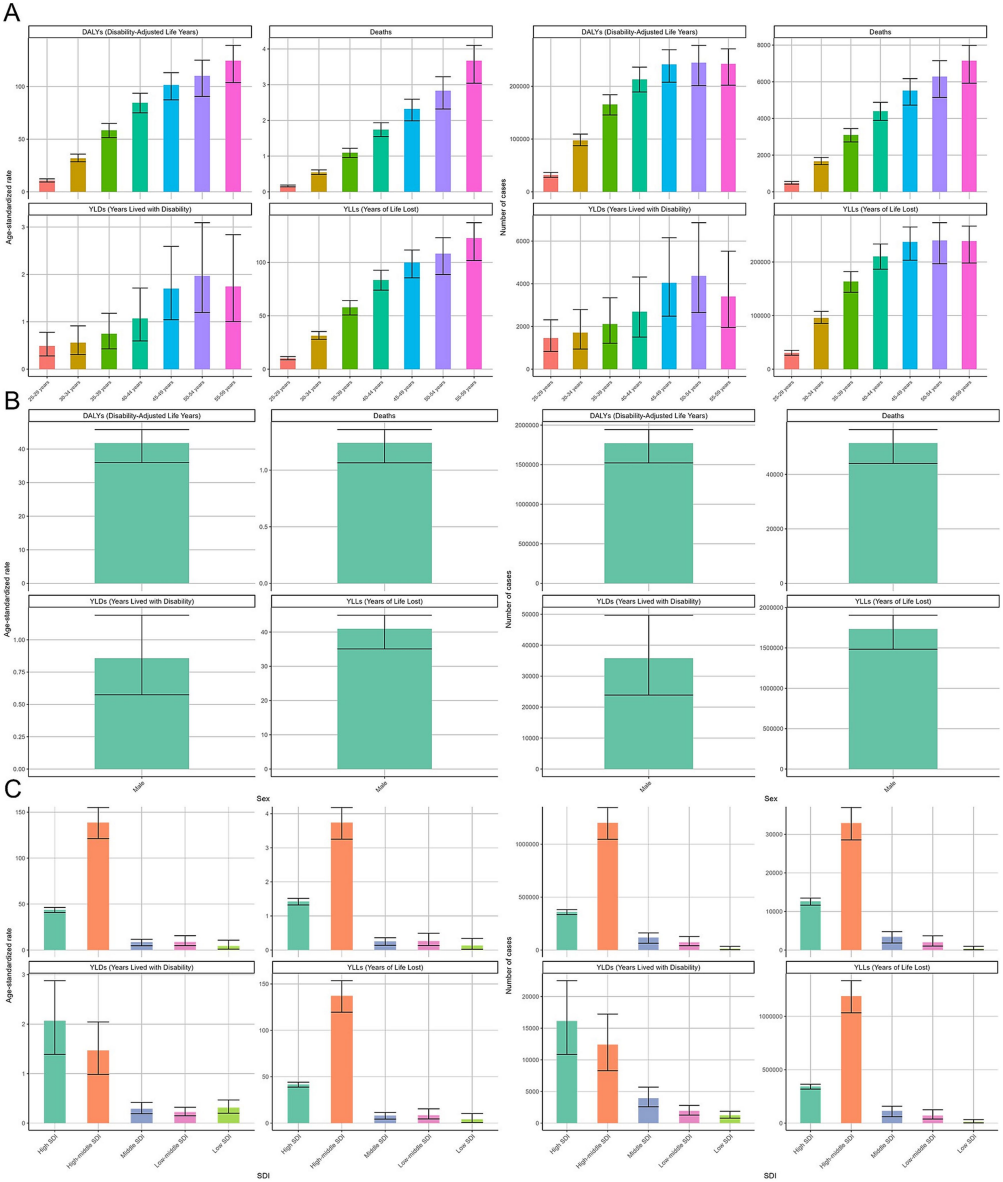
Global burden of alcoholic cardiomyopathy (ACM) in 2021, stratified by region, sex, and socio-demographic index (SDI). **(A)** Age-standardized rates and absolute numbers of disability-adjusted life years (DALYs), deaths, years lived with disability (YLDs), and years of life lost (YLLs) across different geographic regions. Higher ACM burden is observed in Eastern Europe, Central Asia, and Latin America. **(B)** Comparison of ACM burden between males and females, showing significantly higher mortality and DALYs in males. **(C)** ACM burden across SDI groups, with the highest disease burden observed in high-middle SDI countries. Error bars represent 95% uncertainty intervals.

The age-standardized DALY rate increases with age, peaking at 55–59 years, with absolute DALYs rising steeply after 40 years. The age-standardized death rate follows a similar pattern, with absolute deaths peaking in 45–59 years, indicating strong age-dependent mortality risk. YLD rates increase with age but show higher variability in older groups. Absolute YLDs suggest a greater ACM-related disability burden in older adults. Standardized YLL rates rise consistently, with absolute YLLs peaking in 45–59 years, highlighting significant premature mortality (Figure 1A; Tables 1–4).

Among males, the age-standardized DALY rate is 41.81 (95% UI: 35.97–45.8) per 100,000, with 1,771,812 (95% UI: 1,521,953–1,943,500) DALY cases. The age-standardized death rate is 1.24 (95% UI: 1.06–1.36) per 100,000, with over 50,000 deaths, confirming ACM’s high fatality. The YLD rate is 0.86 (95% UI: 0.57–1.19) per 100,000, with 35,784 (95% UI: 23,885–49,677) cases, indicating substantial non-fatal disease burden. YLL rates mirror DALYs at 40.95 (95% UI: 35.08–44.92) per 100,000, with 1,736,028 (95% UI: 1,484,390–1,905,162) cases, reinforcing premature mortality impact (Figure 1B; Tables 1–4).

The high-middle SDI group bears the highest burden, with a DALY rate of 138.73 (95% UI: 121.21–155.03) per 100,000 and 1,201,785 (95% UI: 1,046,653–1,346,640) cases. The death rate is 3.74 (95% UI: 3.25–4.18) per 100,000, with most ACM-related deaths in this SDI group. The highest YLD rate is in high SDI, while both high and high-middle SDI groups have the most absolute YLD cases. YLL rates peak in high-middle SDI at 137.26 (95% UI:119.57–153.56) per 100,000, with 1,189,376 (95% UI:1,032,936–1,334,116) years of life lost annually, emphasizing substantial premature mortality in these regions (Figure 1C; Tables 1–4).

**Figure 2:**
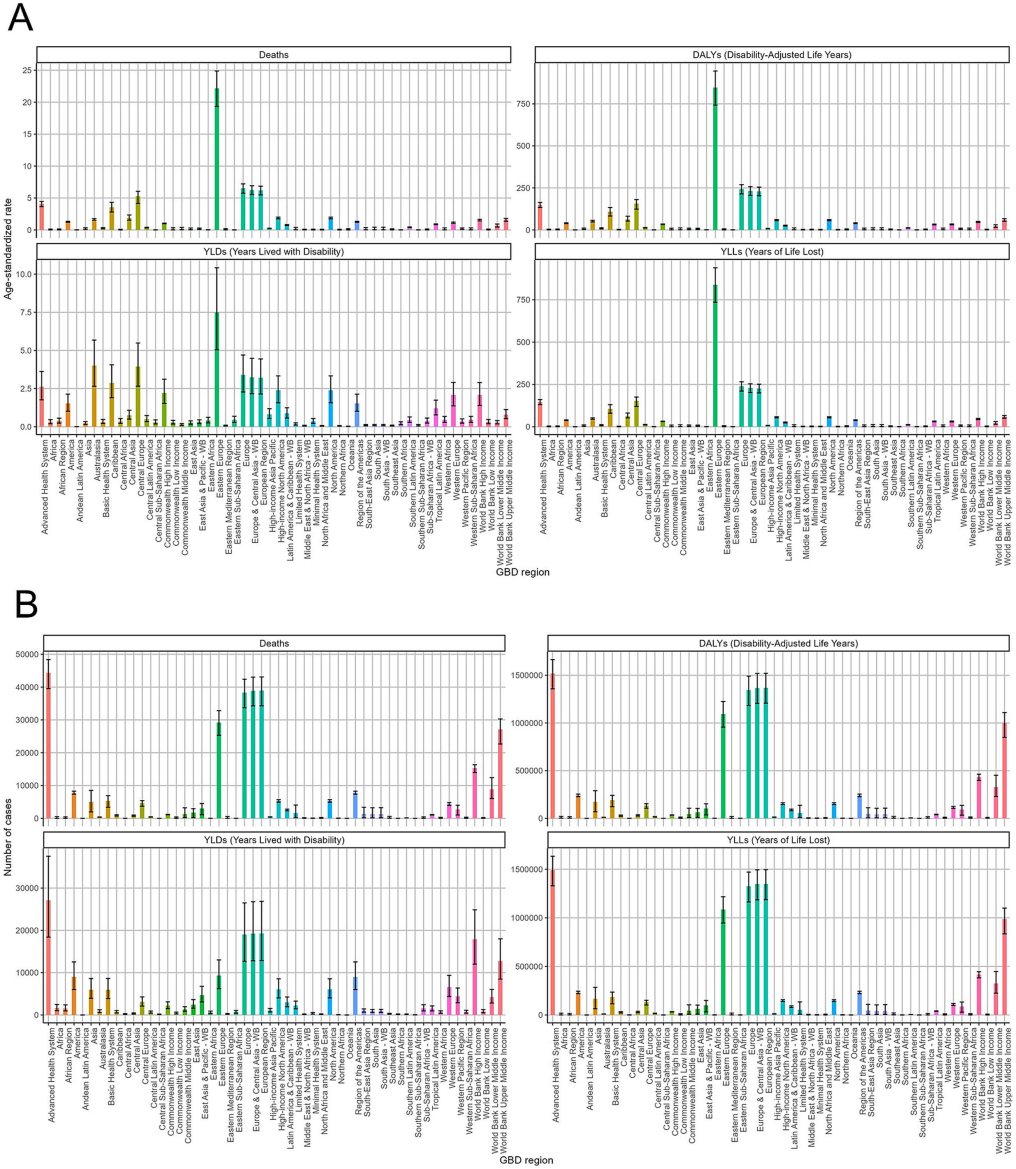
Regional distribution of alcoholic cardiomyopathy (ACM) burden in 2021 across Global Burden of Disease (GBD) regions. **(A)** Age-standardized rates of deaths, disability-adjusted life years (DALYs), years lived with disability (YLDs), and years of life lost (YLLs) per 100,000 population across GBD regions. The highest burden is observed in Eastern Europe, Central Asia, and certain regions of Latin America. **(B)** Absolute number of ACM-related deaths, DALYs, YLDs, and YLLs by GBD region. Eastern Europe and Russia exhibit the highest absolute burden, while other regions show lower but variable impacts. Error bars represent 95% uncertainty intervals.

Across the 45 GBD regions, the age-standardized death rate varies significantly across regions, with Eastern Europe showing the highest rates, exceeding 22.18 (95% UI:19.32–24.87) per 100,000. The absolute number of deaths follows a similar trend, with the highest burden observed in Eastern Europe (29,211, 95% UI: 25,310–32,820) and some Central Asian regions. The age-standardized DALY rate is highest in Eastern Europe, Central Asia, and select regions in Latin America, exceeding 846.38 (95% UI:742.3–945.28) per 100,000. The absolute number of DALYs shows Eastern Europe, Russia, and some Latin American regions as the most affected (1,366,595, 95% UI: 1,205,981–1,519,518). The YLD rates are relatively low compared to other metrics but are highest in Eastern Europe (7.5, 95% UI: 5.05–10.41) and Australasia (4, 95% UI: 2.65–5.68). The absolute YLD burden follows a similar trend, indicating ACM contributes more to premature mortality than disability in most regions. Standardized YLL rates are highest in Eastern Europe (838.88, 95% UI:734.94–938.89), reflecting the substantial premature mortality burden. Absolute YLL counts confirm Eastern Europe, Russia, and some Latin American countries as having the greatest number of years of life lost due to ACM (Figure 2).

**Figure 3:**
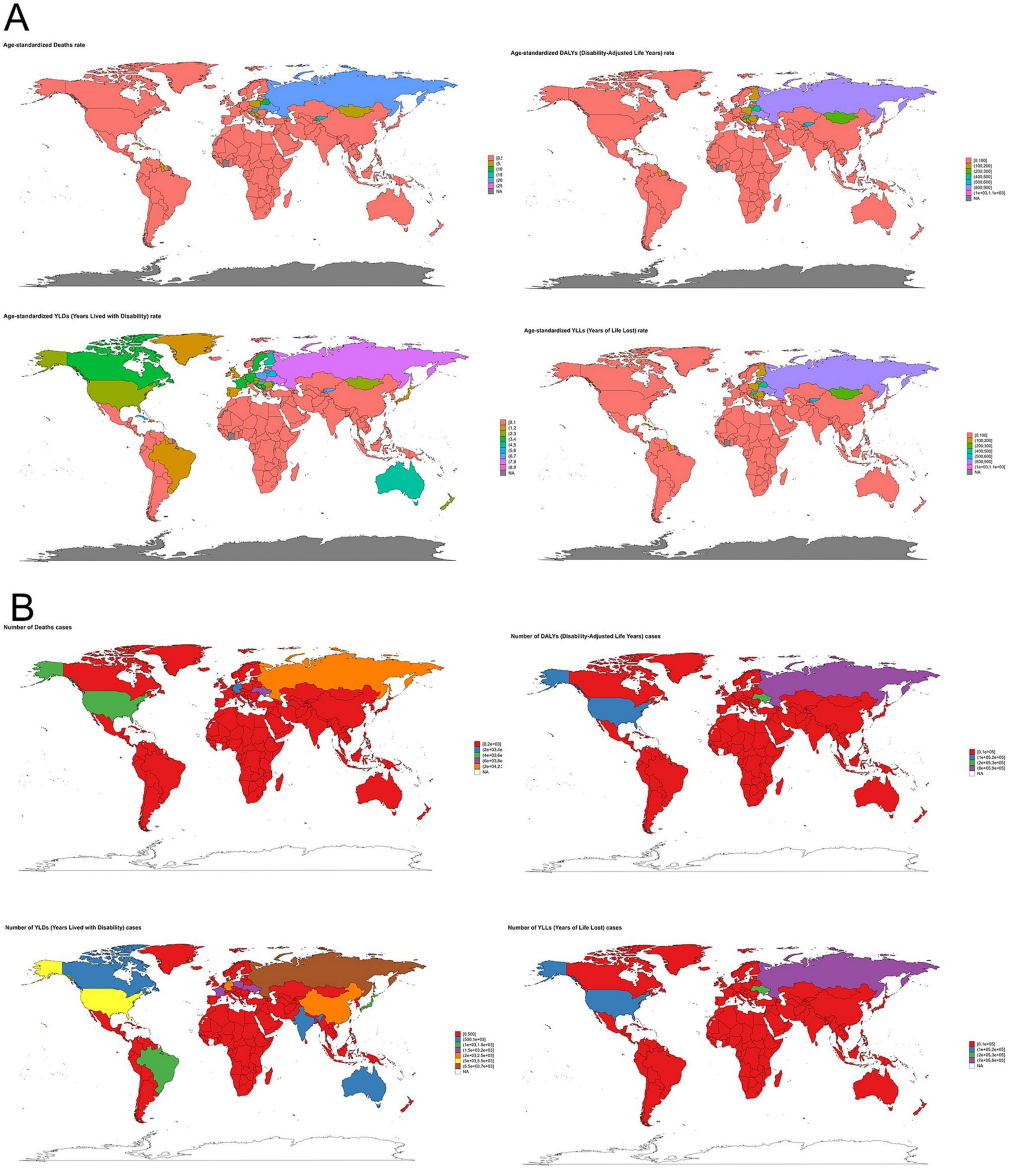
Global distribution of alcoholic cardiomyopathy (ACM) burden in 2021. **(A)** Age-standardized rates of ACM-related deaths, disability-adjusted life years (DALYs), years lived with disability (YLDs), and years of life lost (YLLs) per 100,000 population across countries. The highest age-standardized mortality and disease burden are observed in Eastern Europe, Central Asia, and parts of Latin America. **(B)** Absolute number of ACM-related deaths, DALYs, YLDs, and YLLs by country. Russia, Eastern Europe, and some Latin American regions show the highest absolute burden, whereas other countries experience lower but variable impacts. Color gradients represent different levels of ACM burden, with darker shades indicating higher values.

The global burden of alcoholic cardiomyopathy (ACM) exhibits significant variation across different regions. In 2021, Latvia recorded the highest age-standardized death rate, reaching 26.94 per 100,000 (95% UI: 22.32–31.92), followed by Ukraine and the Russian Federation. In terms of absolute numbers, the Russian Federation had the highest number of ACM-related deaths, totaling 21,394 (95% UI: 18,277–23,984). Similarly, the highest age-standardized DALY rate was observed in South Latvia, at 1,048.76 per 100,000 (95% UI: 877.69–1,232.09), with Ukraine and the Russian Federation ranking next. Conversely, Iraq reported the lowest age-standardized rates for both deaths and DALYs, indicating a significantly lower ACM burden in that region. When considering years lived with disability (YLDs), Latvia again reported the highest age-standardized YLD rate, reaching 8.22 per 100,000 (95% UI: 5.45–12.1), followed by the Russian Federation and Ukraine. In absolute terms, the Russian Federation had the highest number of YLDs, amounting to 6,777 cases (95% UI: 4,559–9,434). A similar pattern was observed for years of life lost (YLLs), with Latvia recording the highest age-standardized YLL rate, at 1,040.54 per 100,000 (95% UI: 870.06–1,223.97). Meanwhile, the Russian Federation had the largest absolute number of YLL cases, reaching 794,022 (95% UI: 683,986–887,284) (Figure 3).

### Temporal trend for ACM-related disease burden from 1990 to 2021

**Figure 4:**
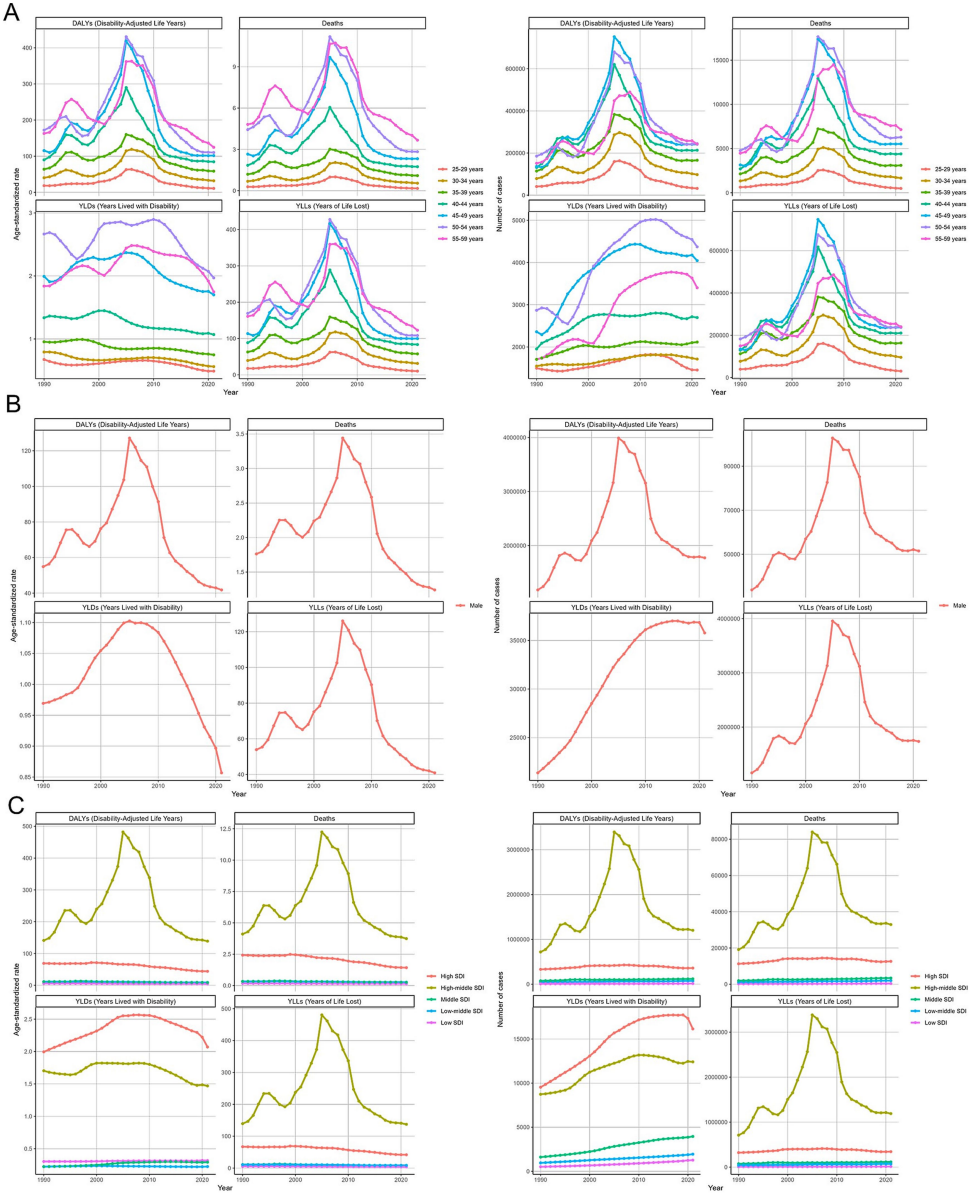
Temporal trends of alcoholic cardiomyopathy (ACM) burden from 1990 to 2021. **(A)** Age-specific trends in disability-adjusted life years (DALYs), deaths, years lived with disability (YLDs), and years of life lost (YLLs) per 100,000 population and absolute numbers. The burden peaks around 2005–2010, with middle-aged groups (45–59 years) experiencing the highest mortality and disease impact. **(B)** ACM burden trends among males, showing a sharp increase until 2010, followed by a gradual decline. Mortality and YLLs remain dominant contributors to disease burden, while YLDs contribute comparatively less. **(C)** Trends stratified by socio-demographic index (SDI) groups, indicating that high-middle SDI countries bear the highest ACM burden, while low-SDI regions exhibit relatively stable but lower levels of disease impact. Lines represent annual estimates, with fluctuations reflecting changes in global alcohol consumption patterns and public health interventions.

The global burden of alcoholic cardiomyopathy ACM increased significantly from 1990 to 2021, peaking around 2005–2010, followed by a gradual decline. Middle-aged adults bore the highest burden, with DALYs, deaths, and YLLs peaking in this age group. The primary driver of ACM burden was premature mortality, while YLDs contributed less to overall disease impact. The trends suggest that alcohol consumption and cardiovascular risks played a major role in early increases, while subsequent declines may reflect improved awareness, interventions, and alcohol control policies. Strengthened public health strategies targeting middle-aged populations are essential to reducing ACM-related mortality (Figure 4A). In Figure 4B, this figure illustrates the temporal trends of ACM burden among males from 1990 to 2021. The left panel shows age-standardized rates, while the right panel presents absolute numbers for DALYs, deaths, YLDs, and YLLs. Both DALYs and deaths increased steadily until peaking around 2010, followed by a significant decline. YLLs display a similar trend, with a sharp rise and subsequent decrease. In contrast, YLDs gradually increased and plateaued, showing a slight decline toward 2021. The burden of alcoholic cardiomyopathy (ACM) from 1990 to 2021 varied significantly across SDI groups. High-middle SDI countries experienced the highest burden, with DALYs, deaths, and YLLs peaking around 2005–2010, followed by a decline. High SDI countries had the highest YLDs, indicating greater disability but lower mortality. Middle, low-middle, and low SDI regions had relatively stable trends with lower ACM burden (Figure 4C).

**Figure 5:**
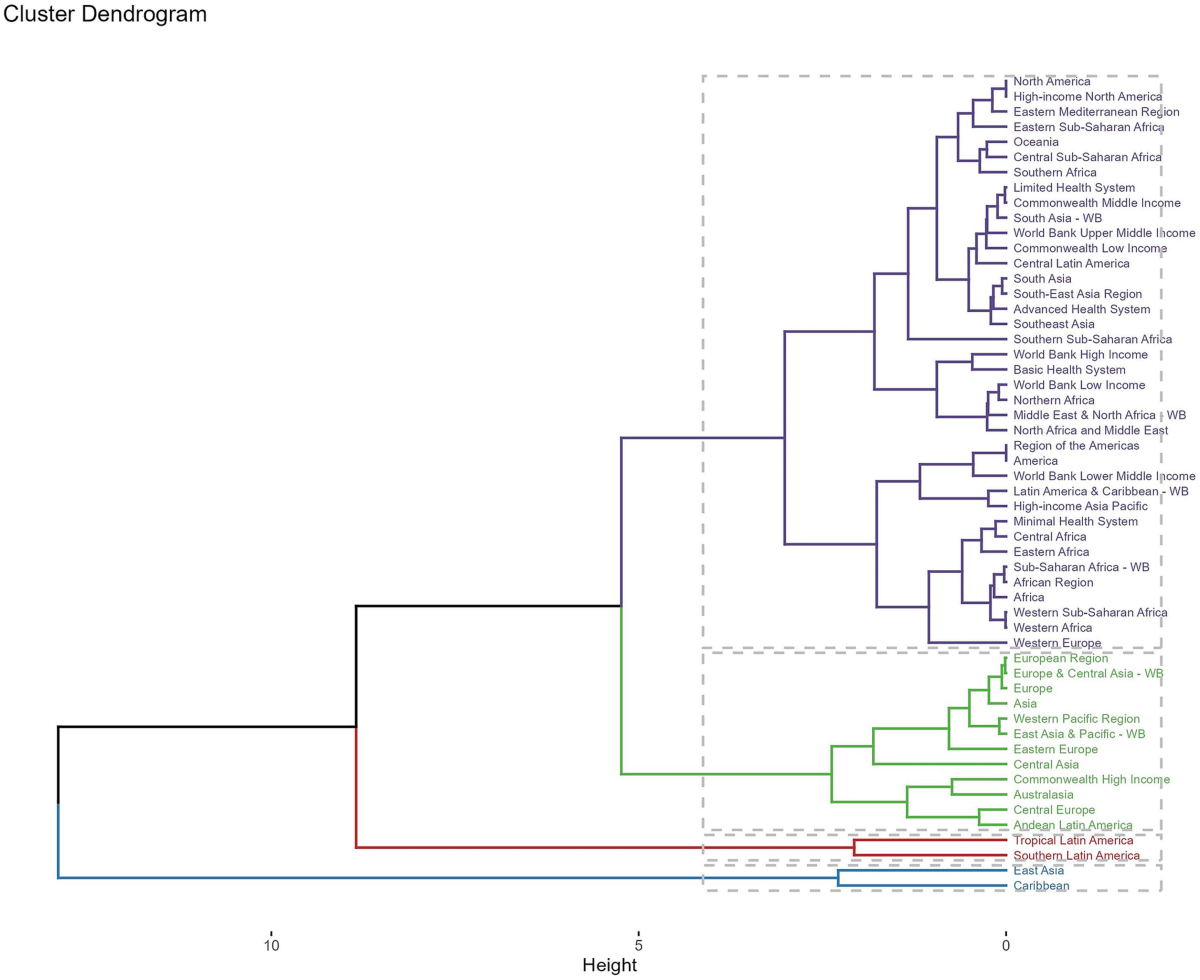
Hierarchical clustering dendrogram of global regions based on the burden of alcoholic cardiomyopathy (ACM). The dendrogram illustrates the clustering of different geographic regions according to their ACM burden using hierarchical clustering analysis. Regions with similar disease burden patterns are grouped together, with branch height representing the degree of dissimilarity. Distinct clusters indicate regional variations, with Latin America, Eastern Europe, and Central Asia forming separate high-burden groups, while Sub-Saharan Africa and South Asia cluster separately, reflecting lower ACM prevalence. High-income regions form another distinct cluster, suggesting a more controlled ACM impact due to healthcare access and policies. This clustering highlights geographic disparities in ACM burden and emphasizes the need for region-specific interventions.

Across GBD regions, the ACM-related disease burden varied. To find regions with similar variation in disease burden, a hierarchical clustering analysis was conducted in this study. The results were shown in Figure 5. The hierarchical clustering dendrogram illustrates regional similarities in the burden of ACM based on GBD data. Regions are grouped based on their ACM burden patterns, with Latin America, Eastern Europe, and Central Asia forming distinct clusters, indicating a higher disease burden. Sub-Saharan Africa and South Asia cluster separately, reflecting lower ACM prevalence. High-income regions cluster together, suggesting a more controlled ACM impact due to healthcare access and policies. This clustering highlights geographic disparities in ACM burden and the need for region-specific interventions.

**Figure 6:**
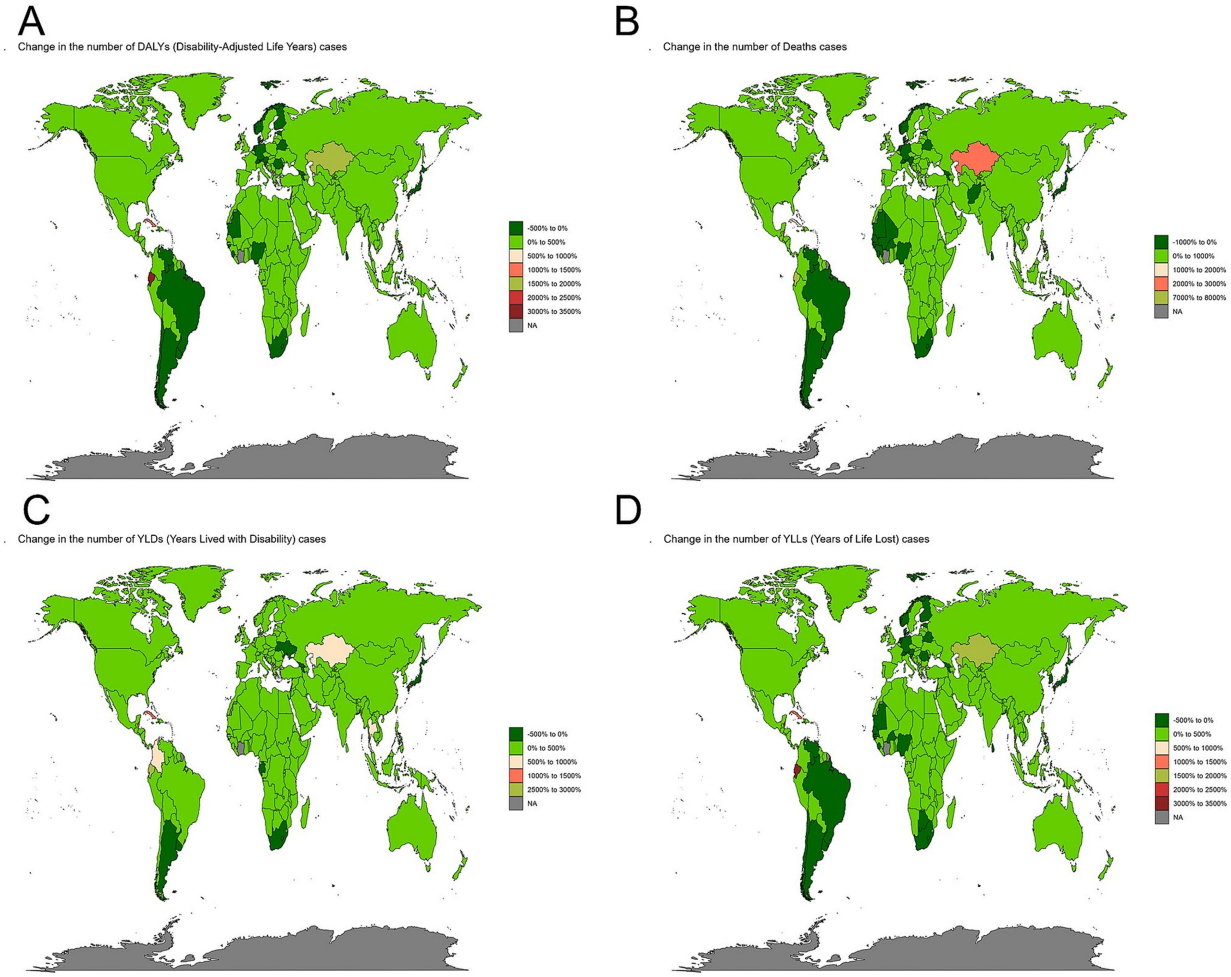
Global changes in alcoholic cardiomyopathy (ACM) burden from 1990 to 2021. **(A)** Change in the number of disability-adjusted life years (DALYs) cases, reflecting the overall ACM disease burden. **(B)** Change in the number of ACM-related deaths, highlighting regions with increasing or decreasing mortality trends. **(C)** Change in the number of years lived with disability (YLDs) cases, indicating trends in the non-fatal burden of ACM. **(D)** Change in the number of years of life lost (YLLs) cases, showing shifts in premature mortality due to ACM. Color gradients represent percentage changes, with darker green indicating reductions, while red and pink areas signify significant increases in ACM burden. Regions experiencing the highest increases are mainly in Central Asia, Eastern Europe, and parts of South America, while some high-income and African regions show declines.

The global burden of ACM from 1990 to 2021, analyzed through DALYs, number of deaths, YLDs, and YLLs, exhibits significant regional disparities. High-income regions, such as North America and Western Europe, show a decline or stabilization across all indicators, likely due to improved healthcare, alcohol control policies, and early interventions. In contrast, South America, Central Asia, and parts of Africa report increasing trends, particularly in deaths and YLLs, indicating a rising burden of premature mortality. YLDs have increased in some regions, suggesting a growing disability impact, while DALYs display mixed trends, with declines in certain areas but surges in others. These findings emphasize the urgent need for targeted public health strategies, including stricter alcohol regulations, early cardiovascular screening, and improved access to medical care. Addressing these regional disparities will be essential to reducing the global ACM burden and improving cardiovascular health outcomes worldwide (Figure 6).

**Figure 7:**
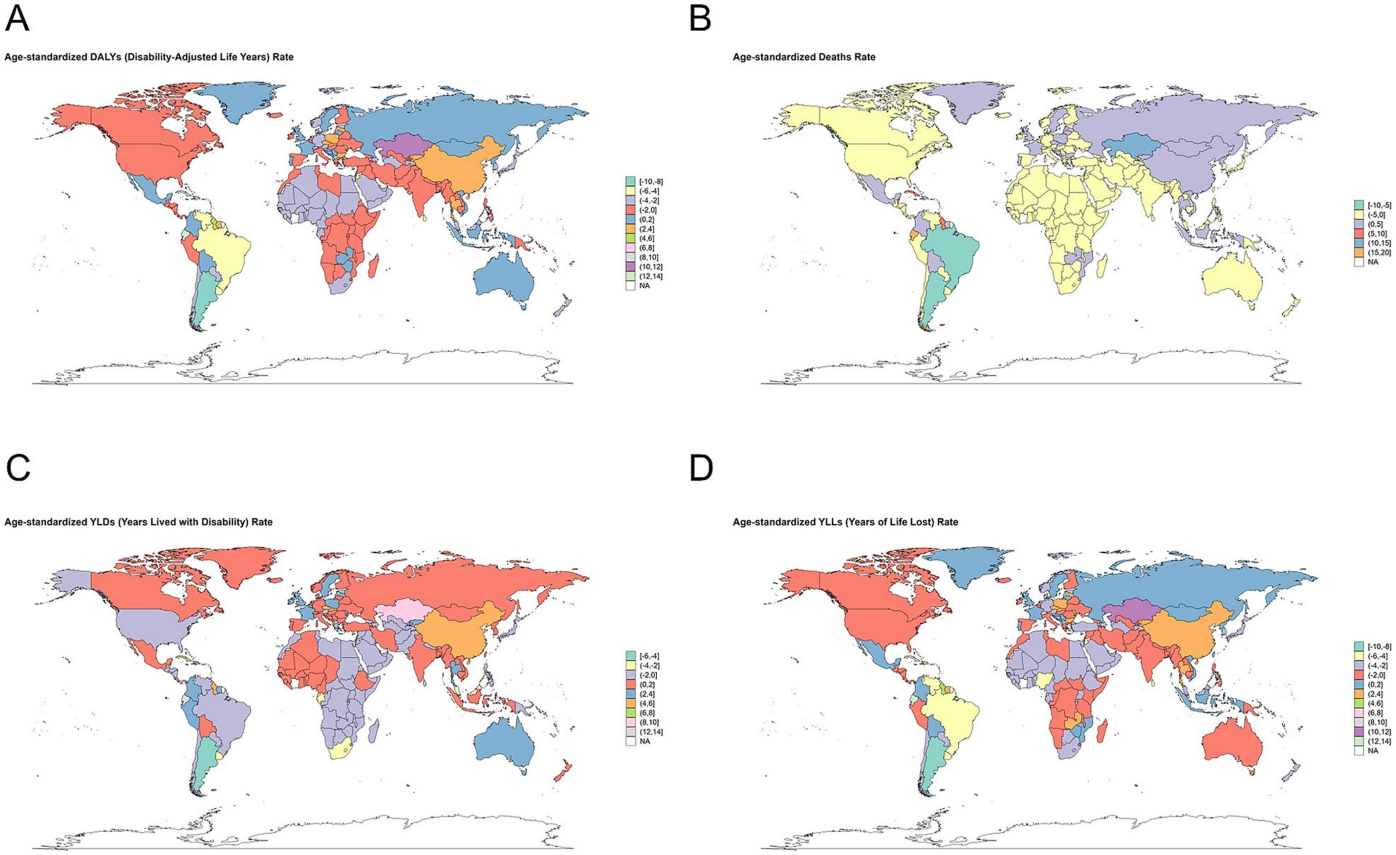
Global distribution of age-standardized alcoholic cardiomyopathy (ACM) burden rates in 2021. **(A)** Age-standardized disability-adjusted life years (DALYs) rate per 100,000 population, representing the overall ACM disease burden. **(B)** Age-standardized death rate per 100,000 population, indicating mortality trends due to ACM. **(C)** Age-standardized years lived with disability (YLDs) rate per 100,000 population, reflecting the non-fatal burden of ACM. **(D)** Age-standardized years of life lost (YLLs) rate per 100,000 population, highlighting premature mortality due to ACM. Color gradients represent different levels of ACM burden, with darker red shades indicating higher rates. The highest burden is concentrated in Eastern Europe, Central Asia, and parts of Latin America, while lower rates are observed in certain high-income and African regions.

The EAPC (Estimated Annual Percentage Change) maps of alcoholic cardiomyopathy (ACM) from 2019 to 2021, covering DALYs, deaths, YLDs, and YLLs, reveal significant global disparities in disease burden trends. North America, Western Europe, and parts of Asia generally show a decline in all indicators, likely due to effective alcohol control policies, improved healthcare, and early interventions. However, Eastern Europe, South America, and parts of Africa exhibit rising trends, particularly in YLLs and deaths, indicating a growing burden of premature mortality. YLDs have increased in several regions, suggesting an increasing disability impact, while DALYs trends are mixed, with some countries experiencing significant surges. These findings emphasize the urgent need for region-specific public health strategies, including stricter alcohol regulations, early cardiovascular screening, and better access to medical care, to mitigate both ACM-related mortality and disability. Addressing these disparities is crucial for reducing the global burden of ACM and improving cardiovascular health worldwide (Figure 7).

### The predicted results from 2022 to 2050

**Figure 8:**
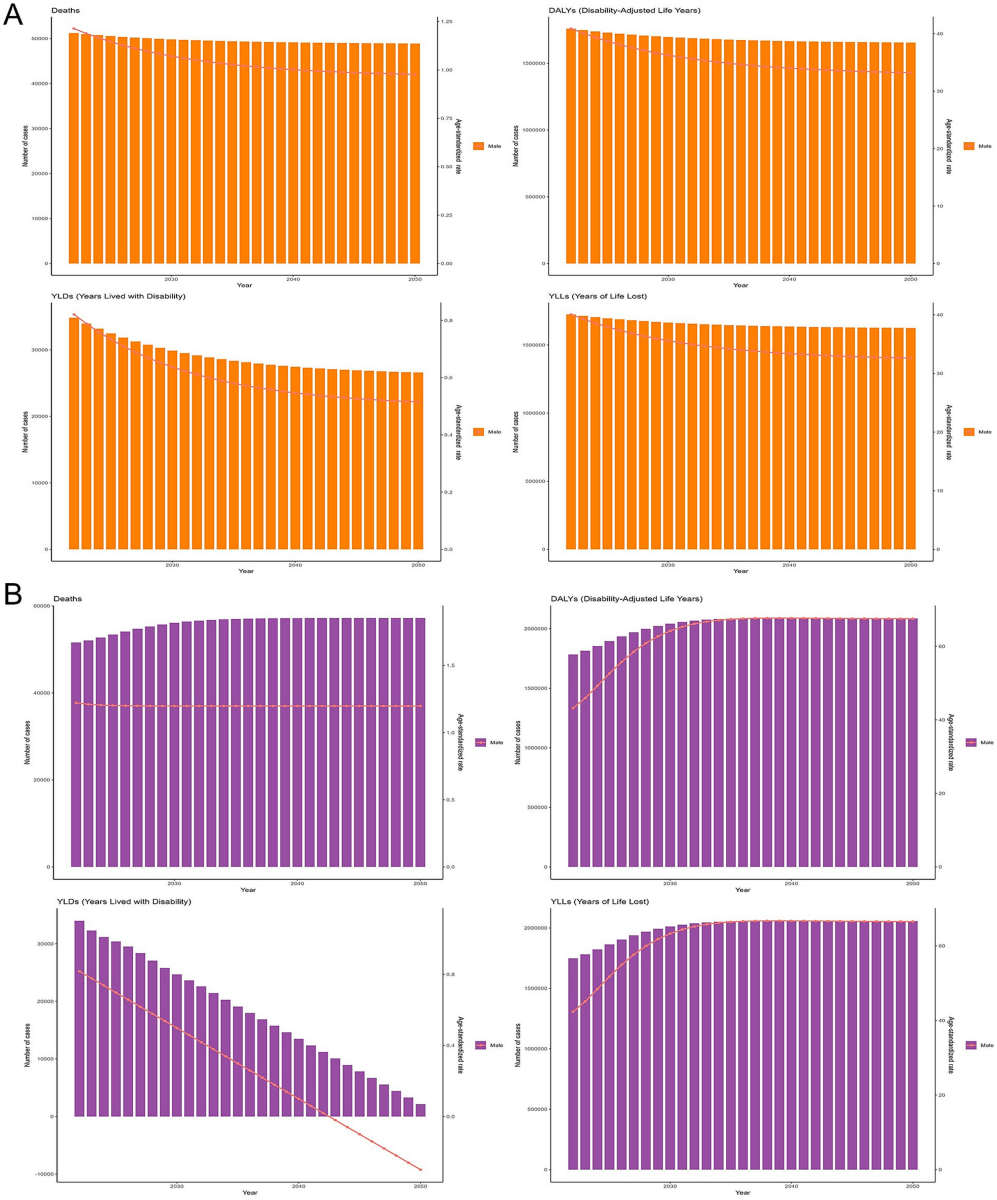
Future projections of alcoholic cardiomyopathy (ACM) burden from 2022 to 2050 using time-series forecasting models. **(A)** Forecasted trends in ACM-related deaths, disability-adjusted life years (DALYs), years lived with disability (YLDs), and years of life lost (YLLs) based on the Autoregressive Integrated Moving Average (ARIMA) model. The projections suggest a stabilization in deaths and DALYs, while YLDs show a gradual decline over time. **(B)** Forecasted ACM burden using the Exponential Smoothing (ES) model. Similar to ARIMA projections, the ES model predicts a plateau in deaths and DALYs but a more pronounced decline in YLDs, reflecting potential improvements in disease management and disability prevention. Bars represent annual estimated cases, and trend lines indicate the projected trajectory. These projections highlight the need for sustained efforts in alcohol control, cardiovascular screening, and healthcare interventions to mitigate ACM burden globally.

The forecasting analysis of alcoholic cardiomyopathy (ACM) burden using the ARIMA and Exponential Smoothing (ES) models provides complementary insights into future trends up to 2050. Both models suggest a potential stabilization or gradual decline in ACM-related mortality, though the ARIMA model projects a more persistent burden in terms of deaths and Years of Life Lost (YLLs), whereas the ES model suggests a gradual decline. DALYs are expected to remain high, with the ARIMA model indicating a plateau, while the ES model projects a slow but steady decline, reflecting ongoing challenges in reducing ACM’s overall health impact. YLDs show a consistent downward trend in both models, suggesting potential improvements in disease management and disability prevention. These projections highlight the need for sustained global efforts in alcohol control, cardiovascular screening, and medical interventions to effectively reduce the long-term burden of ACM and prevent premature deaths while addressing disability concerns (Figure 8).

### Out-of-sample performance of ARIMA and exponential smoothing models

To evaluate the predictive accuracy of the ARIMA and Exponential Smoothing (ES) models, we performed out-of-sample validation using observed GBD data from 2016 to 2021. Table 5 summarizes the performance metrics, including Mean Absolute Percentage Error (MAPE) and Root Mean Squared Error (RMSE), for four key indicators: DALYs, deaths, YLLs, and YLDs, in both absolute values and age-standardized rates. Overall, the ES model demonstrated consistently superior performance across all indicators, with MAPE values <2% and minimal RMSE. In contrast, ARIMA showed high error rates, particularly for ASRs, with MAPE values exceeding 36,000% in DALYs and over 2 million in YLDs, indicating model instability or poor fit in this context. These results suggest that ES is more robust for short-term projections of relatively stable epidemiological metrics such as ACM burden, while ARIMA may be less reliable in this domain. Detailed comparison results are presented in Table 5.

**Table 5:** Forecast accuracy comparison for ACM burden indicators (2016–2021).

Measure	Metric	ARIMA MAPE (%)	ARIMA RMSE	ES MAPE (%)	ES RMSE
DALYs	Number	99.22	6815237.00	1.75	155219.00
	Rate	36778.65	52970.29	0.81	1.53
Deaths	Number	70.30	126416.29	1.84	4201.99
	Rate	1403828.29	53110.51	0.84	0.04
YLDs	Number	53.08	60320.93	1.04	1605.00
	Rate	2167800.11	53111.85	0.39	0.01
YLLs	Number	99.21	6701942.27	1.76	154372.48
	Rate	37416.98	52972.74	0.82	1.52

DALYs, disability-adjusted-life-years; YLDs, years lived with disability’s; YLLs, years of life lost; ARIMA, autoregressive integrated moving average; ES, exponential smoothing; MAPE, mean absolute percentage error; RMSE, root mean squared error.

## Discussion

ACM remains a significant global health concern, particularly among middle-aged males, as demonstrated by this study’s comprehensive analysis using the Global Burden of Disease (GBD) dataset from 1990 to 2021. Our findings highlight substantial disparities in ACM burden across different regions, with the highest mortality and disability-adjusted life years (DALYs) observed in Eastern Europe, Central Asia, and Latin America. The study also provides future projections using ARIMA and ES models, suggesting potential stabilization or a gradual decline in ACM-related mortality, but continued high disease burden in some regions. This research advances the understanding of ACM’s epidemiological trends, addressing gaps in global burden estimation and forecasting, and underscores the urgent need for targeted public health interventions and policy reforms to mitigate ACM’s impact.

This study’s major findings align with existing literature on the detrimental effects of chronic alcohol consumption on cardiovascular health. Prior research has established that ACM results from prolonged alcohol toxicity (13), leading to myocardial fibrosis (14), mitochondrial dysfunction (15), and increased oxidative stress (16), ultimately progressing to heart failure. The observed peak in ACM burden around 2005–2010, followed by a gradual decline, is consistent with global trends in alcohol consumption and cardiovascular disease control measures (17). A significant driver of this trend appears to be the introduction of alcohol taxation policies and public health awareness campaigns in high-income countries (18). However, the persistence of high ACM burden in low- and middle-income regions suggests that these interventions remain insufficient or ineffective in some settings, possibly due to differences in healthcare accessibility, socio-economic status, and cultural norms surrounding alcohol use. Furthermore, our study highlights the increasing disability burden associated with ACM, as reflected in the rising years lived with disability (YLDs) in specific regions. This indicates that although mortality may have declined, the long-term sequelae of ACM continue to affect patients, calling for more effective management strategies.

Interestingly, our findings show that the highest age-standardized burden of ACM was observed in high-middle SDI countries, rather than in high or low SDI regions. This may reflect a combination of sustained high levels of alcohol consumption, limited access to early cardiovascular screening, and underdeveloped chronic disease management systems in these transitional economies. In contrast, high-SDI countries have more advanced healthcare systems and stronger alcohol control policies, which may mitigate ACM-related mortality and disability. Meanwhile, the burden in low-SDI regions might be underestimated due to underreporting and limited diagnostic capacity, highlighting the need for improved data infrastructure.

Although our study focused on middle-aged males, growing evidence suggests that women may exhibit increased susceptibility to alcohol-related cardiac damage. While our study focused on middle-aged males due to their disproportionately higher absolute burden of ACM, it is important to acknowledge the growing body of evidence suggesting that females may be more susceptible to alcohol-induced cardiac damage. Despite generally consuming less alcohol than men, women appear to develop alcoholic cardiomyopathy and other alcohol-related end-organ diseases at lower levels of exposure (5). This heightened vulnerability may be attributed to sex-specific differences in alcohol metabolism, hormonal factors, and myocardial sensitivity to ethanol toxicity. Consequently, future studies should aim to incorporate sex-specific analyses and explore the underlying biological mechanisms, which may help guide gender-sensitive prevention and intervention strategies. Although sex-disaggregated data are available in the GBD dataset, our study was designed to focus on middle-aged males due to their disproportionately higher absolute burden of ACM across all global regions. However, incorporating sex-specific analyses in future research would provide a more comprehensive understanding of the global burden, especially given emerging evidence of increased female susceptibility to alcohol-related cardiac damage. Future GBD and epidemiological studies should stratify ACM trends by sex to enhance prevention strategies.

In addition to the biological and sex-specific considerations, another methodological issue emerged from our analysis—namely, the markedly low estimates of YLDs in ACM. Despite this upward trend, the YLDs attributable to ACM remain significantly lower than YLLs and total DALYs across most regions. While this may partly reflect the true nature of ACM as a disease with high acute mortality, it is also likely influenced by limitations in disability data capture. In many settings, particularly in low-resource countries, subclinical or early-stage ACM cases may go undiagnosed due to limited access to echocardiography or specialist care. Furthermore, the disability weights assigned in the GBD framework may not fully capture the nuanced impact of chronic alcohol-related cardiac dysfunction on patient’ quality of life. These factors may collectively contribute to underestimation of the true non-fatal burden of ACM.

The implications of these findings are substantial for public health planning and disease prevention strategies. The significant regional disparities identified in our analysis emphasize the need for tailored interventions that consider local socio-economic and healthcare factors. Countries with high ACM burden should prioritize alcohol control policies, including taxation, advertising restrictions, and screening programs for early detection of alcohol-related heart disease. In addition, the rising YLDs underscore the need for enhanced rehabilitation and long-term management of ACM patients to improve quality of life and reduce disability burden. To effectively mitigate the rising burden of ACM, multilevel public health interventions are urgently needed. At the local and national levels, strategies such as increasing alcohol taxes, restricting marketing and sales of alcoholic beverages, implementing community-level awareness campaigns, and incorporating routine alcohol-use screening in primary healthcare settings can significantly reduce alcohol consumption and improve early detection. Internationally, collaboration through global health organizations such as the WHO can facilitate data sharing, promote standardized diagnostic criteria, and support the development of best-practice guidelines for managing alcohol-related cardiac diseases. Capacity-building in low- and middle-income countries, including training healthcare professionals and improving access to cardiovascular care, is essential to reduce global disparities in ACM outcomes (18). In projecting the future burden of ACM, we employed both ARIMA and exponential smoothing (ES) models to ensure robust time series forecasting. ES outperformed ARIMA in our validation, with a lower mean absolute percentage error (MAPE < 2%) and RMSE across all indicators. Despite ARIMA’s higher error rates in our validation, its inclusion provides an alternative projection under more volatile trend assumptions. This dual-model approach offers a broader range of plausible future scenarios and enhances the credibility of our long-term estimates to 2050. Future research should focus on identifying effective treatment strategies and public health interventions that can mitigate the impact of ACM, particularly in regions experiencing increasing disease burden. Collectively, these interventions may help curb the increasing global burden of ACM.

Despite its strengths, this study has several limitations. First, the reliance on the GBD dataset, while comprehensive, may be subject to data quality issues in certain regions, particularly those with limited healthcare infrastructure and reporting systems. Second, the study’s forecasting models, though robust, depend on historical trends and may not fully capture future policy changes, healthcare advancements, or shifts in alcohol consumption patterns. Third, while this study highlights regional and demographic disparities in ACM burden, it does not explore individual-level risk factors such as genetic predisposition, dietary influences, or co-existing metabolic conditions, which may contribute to variations in ACM prevalence and severity. Another limitation of this study is the lack of a standardized definition of alcoholic cardiomyopathy across different regions and healthcare systems, which may result in under- or over-reporting of cases. Moreover, the diagnosis of ACM can be confounded by overlapping clinical features with other forms of dilated cardiomyopathy, especially in patients with multiple cardiovascular risk factors. These challenges may affect the accuracy and comparability of the estimates derived from GBD data (1). Moreover, the GBD dataset does not incorporate country-level alcohol policy implementation or changes in alcohol consumption patterns directly into ACM-specific burden estimates. As such, temporal trends may not fully reflect the impact of interventions such as taxation, advertising bans, or alcohol sale restrictions. The absence of these data may limit interpretation of causality in burden fluctuations, particularly in regions with recent aggressive alcohol control measures. GBD estimates may be less reliable in regions such as Sub-Saharan Africa, Central Africa, and parts of Southeast Asia, where vital registration systems are incomplete or absent. These limitations may skew cross-regional comparisons and cluster-based analyses, potentially underestimating true burden or misclassifying regional patterns. Addressing these limitations in future studies will enhance the accuracy and applicability of ACM burden assessments and intervention strategies.

In conclusion, this study provides a comprehensive epidemiological assessment of ACM using the GBD 1990–2021 dataset, offering critical insights into temporal trends, regional disparities, and future projections of disease burden. Our findings emphasize the ongoing and substantial impact of ACM, particularly in middle-aged males, and highlight the need for targeted interventions to reduce both mortality and disability associated with this condition. Strengthened alcohol control measures, improved access to cardiovascular healthcare, and expanded rehabilitation services for ACM patients will be essential in mitigating its long-term consequences. Future research should aim to refine burden estimation methodologies, identify effective prevention and treatment strategies, and address the broader socio-economic determinants of alcohol-related cardiovascular disease. By implementing evidence-based policies and interventions, we can work toward reducing the global burden of ACM and improving cardiovascular health outcomes worldwide.

## Data Availability

The datasets presented in this study can be found in online repositories. The names of the repository/repositories and accession number(s) can be found in the article/supplementary material.
